# Antenatal care and women’s decision making power as determinants of institutional delivery in rural area of Western Ethiopia

**DOI:** 10.1186/s13104-015-1708-5

**Published:** 2015-12-11

**Authors:** Tesfalidet Tekelab, Birhanu Yadecha, Alemu Sufa Melka

**Affiliations:** College of Medical and Health Sciences, Wollega University, P.O.Box 395, Nekemte, Ethiopia

**Keywords:** Institutional delivery, Antenatal care, Decision making, Rural area

## Abstract

**Background:**

Delivery by skilled birth attendance serves as an indicator of progress towards reducing maternal mortality. In Ethiopia, the proportions of births attended by skilled personnel were very low 15 % and Oromia region 14.7 %. The current study identified factors associated with utilization of institutional delivery among married women in rural area of Western Ethiopia.

**Methods:**

A community based cross-sectional study was employed from January 2 to January 31, 2015 among mothers who gave birth in the last 2 years in rural area of East Wollega Zone. A multi-stage sampling procedure was used to select 798 study participants. A pre-tested structured questionnaire was used to collect data and female high school graduates data collectors were involved in the data collection process. Bivariate and multivariable logistic regression model was fit and statistical significance was determined through a 95 % confidence level.

**Results:**

The study revealed that 39.7 % of the mothers delivered in health facilities. Age 15–24 years (AOR 4.20, 95 % CI 2.07–8.55), 25–34 years (AOR 2.21, 95 % CI 1.32–3.69), women’s educational level (AOR 2.00, 95 % CI 1.19–3.34), women’s decision making power (AOR 2.11, 95 % CI 1.54–2.89), utilization of antenatal care (ANC) during the index pregnancy (AOR 1.56, 95 % CI 1.08–2.23) and parity one (AOR 2.20, 95 % CI 1.10–4.38) showed significant positive association with utilization of institutional delivery.

**Conclusion and recommendation:**

In this study proportion of institutional delivery were low (39.7 %). Age, women’s literacy status, women’s decision making power, ANC practice and numbers of live birth were found important predictors of institutional delivery. The findings of current study highlight the importance of boosting women involvement in formal education and decision making power. Moreover since ANC is big pillar for the remaining maternal health services effort should be there to increase ANC service utilization.

**Electronic supplementary material:**

The online version of this article (doi:10.1186/s13104-015-1708-5) contains supplementary material, which is available to authorized users.

## Background

Pregnancy and childbirth are generally times of joy for parents and families. The enabling environment for safe motherhood and childbirth depends on the care and attention provided to pregnant women and newborns by the expertise of skilled birth attendant and the availability of adequate health care facilities, equipment, and medicines and emergency care when needed [1].

Globally, the maternal mortality ratio (MMR) has fallen by 45 % between 1990 and 2013. There were an estimated 289,000 maternal deaths in 2013. Developing countries account for 99 % of the global maternal deaths with sub Saharan Africa region alone accounting for 62 %. Ethiopia’s MMR, 676 per 100,000 live births, is among the highest in the world. Together with only five other countries, namely: India, Nigeria, Pakistan, Afghanistan, and the Democratic Republic of Congo, Ethiopia contributes about 50 % to the total global burden [2, 3].

Maternal health care service utilization is important for the improvement of both maternal and child health and reduces maternal and child mortality and improves the reproductive health of the women. Improving maternal and child health requires increasing the percentage of women giving birth in health institutions with the assistance of trained staff, which is the central goal of the safe motherhood and child survival movements [4–7].

Proven and cost effective interventions can decrease neonatal deaths by three-quarters. Many of these interventions can be delivered through maternal health care service utilization including skilled birth attendant and health facility delivery which represent major chances to reach mothers and their newborn with lifesaving services that often bring better health outcome and developmental benefits. Improved care at birth or delivery is also the most effective strategy for reducing newborn mortality and stillbirths [8].

Delivery by skilled birth attendance serves as an indicator of progress towards reducing maternal mortality [9].

In developing regions overall, the proportion of deliveries attended by skilled birth attendant was 65 % in 2010. The regions with the highest maternal mortality, sub-Saharan Africa and Southern Asia, are also those with the lowest coverage of births attended by skilled birth attendants less than half. There have been important improvements in maternal health and reduction in maternal deaths, but progress is still slow. Large disparities still exist in providing pregnant women with antenatal care (ANC) and skilled assistance during delivery. Poor women in remote areas are least likely to receive adequate care during childbirth. This is especially true for regions where the number of skilled birth attendant remains low and maternal mortality high particularly in sub-Saharan Africa [10, 11].

According to the 2014 Ethiopia Demographic and Health Survey (EDHS), the proportions of births attended by skilled personnel were very low 15 % and Oromia region 14.7 % [12]. In the study area information is lacking on the issue. Hence, this study was conducted to determine the prevalence of institutional delivery service utilization and associated factors in rural area of East Wollega Zone, Western Ethiopia.

## Methods

### Study design, setting and participants

A community-based cross-sectional study was carried out from January 2 to January 31, 2015 among mothers who gave birth in the last 2 years in rural area of East Wollega Zone, Oromia Region, West Ethiopia. East Wollega Zone is one of the zones of Oromia Regional state with a population of 1,230,402 among which 614,761 are males and 615,641 are females. Majority of the population live in rural areas 86 % (1,061,120). Nekemte is the capital city of the zone which is located 331 km west of Addis Ababa with a population of 76,817 (male 39,167 and female 37,650) [13]. The source population was all married women aged 15–49 years who gave at least one birth in the last 2 years preceding the survey. Study populations were randomly selected married women aged 15–49 years who gave at least one birth in the last 2 years preceding the survey. Women who were critically ill could not provide informed consent were excluded from the study.

### Sample size and sampling techniques

The sample size was determined using a formula for estimation of single population proportion with the assumption of 95 % confidence interval (CT), margin of error 5 % and taking 61.6 % institutional delivery prevalence of Holeta town, central Ethiopia [14] and a design effect of 2. To avoid the effect of the design that decreases the representativeness of the study we used design effect. To compensate the non-response rate, 10 % of the determined sample was added up on the calculated sample size and the final sample size was 801.

A multi-stage sampling technique was employed for the selection of the sampling units. First, six districts were selected from 18 districts found in East Wollega Zone. Then 10 rural kebeles (lower administrative level) were randomly selected from a list of all kebeles found in the six districts. The calculated sample size was proportionally allocated to each kebeles based on the number of married women who gave birth in the past 2 years. Then picking a house randomly for the initial household from each kebele, the final households with married women were selected using systematic sampling from the existing sampling frame of households which were identified through census prior to data collection. Finally, eligible study subjects were interviewed from each selected households.

### Data collection procedures

Pre-tested structured questionnaires were adapted from different literature [3, 12, 14–18] (Additional file 1). The questionnaires were prepared in English, translated into Afan Oromo (regional language), and then retranslated back to English by people who are proficient in both languages to maintain the consistency of the questionnaires. To administer the structured questionnaires, 12 female high school graduates were selected from the study area. Training was given for 3 days about the objective, relevance of the study, confidentiality of information, respondent’s rights, informed consent and techniques of interview. Six supervisors who have second degree oversaw the data collection procedures. All field questionnaires were reviewed each night and issues that arose during data collection were addressed in morning sessions.

### Data processing and analysis

Data were cleaned and entered into a computer using Epi-Info window version 6.5 statistical programs. The data were then exported to SPSS windows version 20.0 for further analysis. The descriptive analyses such as proportions, percentages, frequency distribution and measures of central tendency were conducted.

Initially, bivariate analysis was performed between dependent variable and each of the independent variables, one at a time. Their odds ratios (OR) at 95 % CI and p-values were obtained. The findings at this stage helped us to identify important associations. Then all variables found to be significant at bivariate level (at *p* < 0.05) were entered into multivariate analysis using the logistic regression model to test the significance of the association.

### Definition of terms

*Skill birth attendant* means having an accredited health professional, including a midwife, doctor, or nurse, who has been trained in the skills needed to needed to manage a normal or uncomplicated pregnancy and childbirth and to support the woman in the immediate postpartum period.

*Institutional delivery* means women who gave birth at health facility (Hospital or health center).

* Home deliveries* means delivery attended by non-skilled birth attendant in this study.

### Ethical considerations

Ethical clearance and permission was obtained from Wollega University Institutional Review Board. Permission was secured from all kebeles through a formal letter. Written informed consent was obtained from each respondent before their interview. The written informed consent was also includes study participants less than 18 years since they were married and minor mature and the consent procedure was approved by ethics committee of Wollega University. Confidentiality of individual client information was ensured by using unique identifiers for the study participants and also limiting access to respondents’ information to the principal investigator and research assistants by storing the completed questionnaires and all documents with participant information in a lockable cabinet.

## Results

### Socio-demographic characteristics

A total of 798 mothers who gave birth in the last 2 years were responded to the questionnaire, making a response rate of 99.6 %. Five hundred eleven (64.0 %) were in the age group of 25–34 years with mean age of 29.5 years (SD ± 5.3 years). Majority (94.2 %) were from Oromo ethnic group. More than half of the respondents (58.0 %) were protestant in religion. Nearly half (47.6 %) respondents can’t read and write, whereas (29.1 %) of the respondents’ husbands had attended grade 1–4. Six hundred thirty five (79.6 %) of the respondent and their husbands (81.1 %) were housewives and farmer, respectively. Their mean monthly income was ETB 841.01. Out of the total mother, 45.0 % owned radio/TV (Table 1).

**Table 1:** Socio demographic characteristics of respondents in rural area of East Wollega Zone, Western Ethiopia, January, 2015

Variables (798)	Number (%)
Age category	
15–24	116 (14.5)
25–34	511 (64.0)
35–44	171 (21.4)
Ethnicity	
Oromo	752 (94.2)
Amhara	40 (5.0)
Tigre	6 (0.8)
Religion	
Protestant	463 (58.0)
Ethiopian orthodox	312 (39.1)
Catholic	3 (0.4)
Muslim	19 (2.4)
Others^a^	1 (0.1)
Educational status of the respondent	
Can’t read and write	380 (47.6)
Can read and write	57 (7.1)
Grade 1–4	154 (19.3)
Grade 5–8	78 (9.8)
Secondary	117 (14.7)
College and above	12 (1.5)
Educational status of the husband	
Can’t read and write	88 (11.0)
Can read and write	132 (16.5)
Grade 1–4	232 (29.1)
Grade 5–8	196 (24.6)
Secondary	114 (14.3)
College and above	36 (4.5)
Occupational status of the respondents	
Housewife	635 (79.6)
Governmental employee	37 (4.6)
Daily laborer	77 (9.6)
Merchant	40 (5.0)
Student	9 (1.1)
Occupational status of the husband	
Farmer	647 (81.1)
Governmental employee	71 (8.9)
Daily laborer	29 (3.6)
Merchant	35 (4.4)
Student	16 (2.0)
Income (ETB)	
<490	200 (25.1)
491–700	216 (27.1)
701–1000	230 (28.8)
>1000	152 (19.0)
Mean	841.01 ETB
Have radio/TV	
Yes	359 (45.0)
No	439 (55.0)

^a^ *Adventist ETB* Ethiopian Birr 1$ = 20.2 ETB

### Obstetric characteristics

Five hundred seven (63.5 %) of the study subjects had two to four children. Majority (92.1 %) of the respondents had no history of abortion. Four hundred ninety four (61.9 %) of the respondents had visited health facilities for ANC purposes during their last pregnancy. Among the mothers who attended ANC, 294 (59.5 %) of them visited health facilities two to three times (Table 2).

**Table 2:** Obstetrics history of respondents in rural area of East Wollega Zone, Western Ethiopia, January, 2015

Variables	Number (%)
Parity (798)	
1	101 (12.7)
2–4	507 (63.5)
≥ 5	190 (23.8)
Abortion in life time	
Yes	63 (7.9)
No	735 (92.1)
ANC visit during last pregnancy	
Yes	494 (61.9)
No	304 (38.1)
Number ANC visit during last pregnancy	
Only one	129 (26.1)
Two to three	294 (59.5)
Four and above	71 (14.4)

### Awareness and place of delivery

From the total study participants 81.0 % of them heard about institutional delivery and 32.7 % of them heard about institutional delivery from health workers. More than three-fourth (76.3 %) of the respondent knew a health problem occur during child birth. Two hundred forty nine (40.9 %) of the respondent mentioned maternal death as a health problem occurring during child birth. Three hundred seventeen (39.7 %) of the respondent gave birth at health facilities and more than half (60.3 %) of respondent gave birth at home (Table 3).

**Table 3:** Awareness and place of delivery of the respondents in rural area of East Wollega Zone, Western Ethiopia, January, 2015

Variables	Number (%)
Ever heard of institutional delivery (798)	
Yes	646 (81.0)
No	152 (19.0)
Source of information on institutional delivery (646)	
Health worker	211 (32.7)
Radio/TV	72 (11.1)
Family/relatives	25 (3.9)
Friends	23 (3.6)
Others	5 (0.8)
Knew a health problem occur during child birth (798)	
Yes	609 (76.3)
No	189 (23.7)
Mention the problem (609)	
Severe bleeding	429 (70.4)
Obstructed labour	98 (16.1)
Fetal death	238 (39.1)
Maternal death	249 (40.9)
Others	25 (4.1)
Place of last 12 month delivery (798)	
Home	481 (60.3)
Health facility	317 (39.7)

### Reason for home delivery

**Fig. 1:**
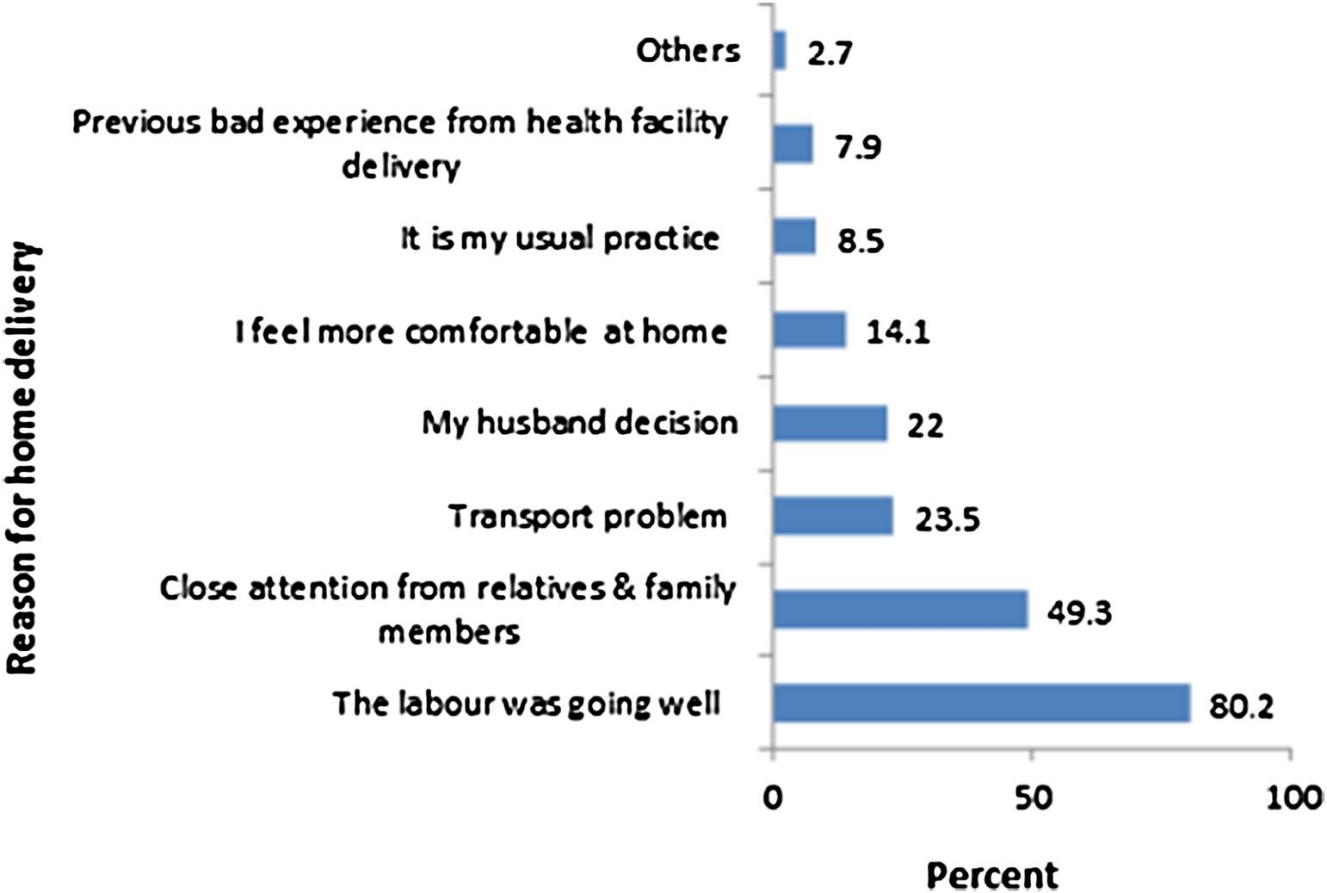
Bar graphs showing reasons for home delivery among mothers in rural area of East Wollega Zone, Western Ethiopia, January, 2015

Among 481 mother who delivered at home 386 (80.2 %) reported that they prefer to deliver at home because the labour was going well (Fig. 1).

### Factors associated with utilization of institutional delivery

In multivariate analysis revealed that mothers with age group of 15–24 years were four times more likely to give birth in the health institution than mothers with age group 35–44 years (AOR 4.20, 95 % CI 2.07–8.55), while mothers aged 25–34 years were two times more likely to have birth at health facility than mothers with age group 35–44 years (AOR 2.21, 95 % CI 1.32–3.69). Those respondents who had secondary school education and above were two times more likely to give birth in health institution than those with primary education and below (AOR 2.00, 95 % CI 1.19–3.34). Those women who decide place of birth by themselves were two times more likely to use institutional delivery than mother whom decision where to give birth made by husband and family members (AOR 2.11, 95 % CI 1.54–2.89). Mother who attended ANC service for the index pregnancy was nearly two times more likely to use institutional delivery than their counterpart (AOR 1.56, 95 % CI 1.08–2.23). Mothers with parity of one had two times the likelihood of utilizing institutional delivery service than mothers with two parity and above (AOR 2.20, 95 % CI 1.10–4.38) (Table 4).

**Table 4:** A multivariate Logistic regression on determinant of institutional delivery in rural area of East Wollega Zone, Oromia region, Western Ethiopia, January, 2015

Characteristics	Utilization of institutional delivery		Crude OR	Adjusted OR
	Yes (%)	No (%)	OR (CI)	OR (CI)
Age category in years				
15–24	80 (69.0 %)	36 (31.0 %)	10.04 (5.77–17.45)	4.20 (2.07–8.55)*
25–34	206 (40.3 %)	305 (59.7 %)	3.05 (1.99–4.68)	2.21 (1.32–3.69)*
35–44	31 (18.1 %)	140 (81.9 %)	1	1
Education of respondents				
Below and primary	234 (35.0 %)	435 (65.0 %)	1	1
Secondary and above	83 (64.3 %)	46 (35.7 %)	3.35 (2.26–4.79)	2.00 (1.19–3.34)*
Education of husband				
Below and primary	243 (37.5 %)	405 (62.5 %)	1	1
Secondary and above	74 (49.3 %)	76 (50.7 %)	1.62 (1.14–2.32)	0.92 (0.53–1.97)
Occupation of respondents				
House wife	233 (36.7 %)	402 (63.3 %)	1.99 (1.40–2.81)	1
Others	84 (51.5 %)	79 (48.5 %)	1	1.42 (0.95–2.14)*
Husband occupation				
Farmer	244 (37.7 %)	403 (62.3 %)	1.55 (1.08–2.21)	1
Others	73 (48.3 %)	78 (51.7 %)	1	0.85 (0.49–1.45)
Decision where to give birth				
Self	187 (50.1 %)	186 (49.9 %)	2.28 (1.71–3.05)	2.11 (1.54–2.89)*
Others	130 (30.6 %)	295 (69.4 %)	1	1
Use of ANC during the index pregnancy				
Yes	240 (48.6 %)	254 (51.4 %)	1	1.56 (1.08–2.23)*
No	77 (25.3 %)	227 (774.7 %)	2.79 (2.04–3.81)	1
Parity				
1	71 (70.3 %)	30 (29.7 %)	7.63 (4.43–13.12)	2.20 (1.10–4.38)*
2–4	201 (39.6 %)	306 (60.4 %)	2.12 (1.45–3.10)	1.24 (0.80–1.92)
≥5	45 (23.7 %)	145 (76.3 %)	1	1
Possessing radio/and TV				
Yes	157 (43.7 %)	202 (56.3 %)	1.36 (1.02–1.80)	1.02 (0.72–1.43)
No	160 (36.4 %)	279 (63.6 %)	1	1

* Statistically significant (p-value < 0.05) *1* Reference category

## Discussion

This community-based study identified the institutional delivery service utilization and associated factors among mothers who gave birth in the last 2 years prior to the study in rural area of East Wollega Zone. The finding of this study showed that 39.7 % of the births were attended in the health institution. The finding was much higher than previous studies in Ethiopia [12, 15, 16]. The difference might be due to the time gap of the studies and there could be improvement in utilizing maternal health care service through health extension workers and governmental commitment to decrease maternal mortality by increasing number of birth attended by skilled birth attendants and health facilities. The finding was lower than study done in Holeta town, central Ethiopia which was 61.6 % [14]. This might be due to the fact that Holeta town is near to the capital city of Ethiopia and could have better opportunities for information and better access to health institutions than rural areas.

The main reasons given by mothers for home delivery was 80.2 % reported that they prefer to deliver at home because the labour was going well, 237 (49.3 %) where close attention from relatives and family members. The finding was similar to previous studies done in Ethiopia [14, 17]. The study highlight the importance of emphasizing on improving women’s awareness on the risk associated with home deliveries.

Institutional deliveries were more common among births to mothers below age 35 years [12]. In this study, mothers whose age category was 15–24 and 25–34 years were more likely to use institutional delivery service than mothers with age group 35–44 years. It is consistent with previous studies [15, 19]. The possible explanation might be older mother may believe that there is less risk to home delivery due to previous knowledge of their pregnancy and delivery.

According to EDHS, highly educated mothers were most likely to have their births assisted by a skilled provider 74 % [3]. Previous studies conducted in developing countries have been found that high education level was associated with a high proportion of facility deliveries. In this study, mothers who attended secondary education and above were two times more likely to give birth in health institution than those mothers who had primary education and below [18, 20–25]. The findings of current study calls for working on improving women’s participation on formal education.

In previous studies mothers autonomy is associated with maternal health care service utilization including place of delivery and delivery assisted by skilled birth attendant [14, 26–31]. In the current study mother for whom the decision on place of delivery made by themselves were two times more likely to give birth in health institution than mother whom decision where to give birth made by others.

Mother who attended ANC service for the index pregnancy was nearly two times more likely to use institutional delivery than their counterpart. This study is similar with studies done elsewhere [17, 32–36]. This could be due to the information mother received during the ANC follow up about the importance of delivering at health institution assisted with skilled birth attendant from the health care provider which could have influenced their decision to deliver with assistant of skilled birth attendant in the health institution.

As parity level increased, women were less likely to give birth in health institution compared with low parity women (women with 1–2 live births) [14, 23, 24, 37, 38]. In this study mothers with parity of one had two times the likelihood of using health facilities than mothers with two parity and above. The possible reason could be mothers who have one child tend to be more concerned about complications than those who have had two or more previous deliveries; hence, they tend to choose skilled birth attendant.

The limitation of this study was the cross-sectional nature of the data that could obscure the causal effect relationships of different factors. In addition it lacks qualitative data.

### Conclusion and recommendation

In this study proportion of institutional delivery were low (39.7 %). Age, women’s literacy status, women’s decision making power, ANC practice and numbers of live birth were found important predictors of institutional delivery. The findings of current study highlight the importance of scaling up of women’s formal education and decision making power. Moreover since ANC is big pillar for the remaining maternal health care services effort should be there to increase ANC utilization.

## Additional file

10.1186/s13104-015-1708-5 Questionnarie.
