# Supplementary material for: Antenatal care and women’s decision making power as determinants of institutional delivery in rural area of Western Ethiopia
Source: BMC Res Notes. 2015 Dec 11;8:769. doi: 10.1186/s13104-015-1708-5 (PMC4676818; doi:10.1186/s13104-015-1708-5)
Supplement: Supplementary file 1 — 10.1186/s13104-015-1708-5 Questionnarie. [file 13104_2015_1708_MOESM1_ESM.docx]

## Information Sheet

**Wollega University**

**College of Medicine and Health Sciences**

Here, We the undersigned, at Wollega University College of Medicine and Health Sciences, currently we were undertook research on a topic entitled assessment of factors affecting utilization of maternal health care services among married women of childbearing age in rural area of East Wollega zone, Oromia region, Ethiopia, 2015.

For this study, you selected as a participant and before getting your consent or permission of your participation, you need to know all necessary information related to the study. Thus, this information detailed as;

- **Objective**: To assess factors affecting utilization of maternal health care services among married women of childbearing age in rural area of East Wollega zone, Oromia region, Ethiopia, 2015.
- **Significance of the study**: The finding of the study will be used by policy makers and program managers as an input to improve service provision on maternal health care.
- **Participants to be included**: All randomly selected married women who gave birth within 12 months
- **Confidentiality:** All information you gave kept confidential and won't be accessible to any third party. Your name won't be registered on the question sheet so that you were not identified.
- **Risks and Benefits of the study**

**Risks:** The study was carried out simply by asking you, the already prepared and structured questions. The procedure didn’t bear any physical or psychological trauma. Furthermore, you didn’t forced to respond to the information you do not know.

**Benefits:** For your participation in the study no payment granted or has no any special privilege to you. But, participating in the study and giving your information to questions asked had great input in efforts to improve utilization of maternal health care service .

- **Consent:** Your participation in the study were based on your willingness. You had the right not to participate from the beginning, or stop any time after starting participation. You didn’t forced to respond to the information you do not know.
- **Name of principal investigator**: Tesfalidet Tekelab

Date:___________ Signature_________________

- **Address of PI:**

Mobile: +251912450760

Mail: [tesfeshtekelab@yahoo.com](mailto:tesfeshtekelab@yahoo.com) or [ttesfalove@gmail.com](mailto:ttesfalove@gmail.com)

## II. Structured Questionnaire English Version

**Wollega University**

**College of Medicine and Health Sciences**

Questionnaire on assessment of factors affecting utilization of maternal health care service among married women of childbearing age in rural area of East Wollega Zone, Oromia region, West Ethiopia.

**Consent form that certify the respondents agreement before the interview**

1. Name of the Kebeles __________________________
2. Questionnaire Identification Number_______________________

**Introduction**

Good morning, Good afternoon [According to its convenience]. My name is ________________________. Now I am collecting data from married women of reproductive age groups(15- 49 years) for the research being conducted to identify factors associated with utilization of maternal health care service , by Mr. Tesfalidet Tekelab ,Mr. Birhanu Yadecha from Wollega University , College of Medicine and Health sciences. You are selected to be one of the participants in the study by chance. The study will be conducted through interview. Your name and other personal identifiers will not be recorded on data collection format and the information that you give us will be kept confidential and will also be used for this study purpose alone. A code number will identify every participant and no names will be used. If a report of the result is published, only summarized information of the total group will appear. The interview takes 30 minutes and is voluntary and you have the right to participate, or not to participate or to refuse at any time during the interview. You will not face any problem if you do not agree to the information to be asked . Your participation on this study helps to improve and identify factors associated with utilization of maternal health care for all married women in rural area of East wollega zone . If you have any questions about this study you may ask me or the principal investigators Mr. Tesfalidet Tekelab (Mobile: +251912450760or E-mail: [tesfeshtekelab@yahoo.com](mailto:tesfeshtekelab@yahoo.com) , [ttesfalove@gmail.com](mailto:ttesfalove@gmail.com) ,

Are you willing to participate in the study?

1. Yes 2. No

- Interviewer who certified that the informed consent has been given verbally from the respondents

Name_____________________________ signature__________________

Date______________________________

- Result

1. Completely collected
2. Refused
3. Partially completed
4. Other (please specify)_________________________________

- Checked by:

Name ______________________ signature_________ Date__________

**Instruction**: For the questions that have alternatives, encircle to the response of the mother. Write appropriate response(s) on the space provided for questions for which alternatives are not given.

**I. Socio-Demographic Characteristics of Respondents**

| **S/N** | **Questions** | **Responses** | **Remark** |
| --- | --- | --- | --- |
| 101 | What is your Age? | _________Years |  |
| 102 | What is your Ethnicity? | 1. Oromo 2. Amhara 3. Tigery 4. Other, specify _________ |  |
| 103 | What is your Religion? | 1. Protestant 2. Orthodox 3. Muslim 4. Catholic 5. Other (Specify) ___________________ |  |
| 104 | Educational level(maternal) | 1. Cannot read and write  2. Able to read and write  3. Primary school – 1- 4 grade  4. Primary school – 5- 8 grade  5. Secondary school  6. College diploma and above |  |
| 105 | What is your occupation?(Maternal) | 1. House wife 2. Government Employed 3. Daily laborer 4. Merchant 5. Student 6. Others[specify]____________ |  |
| 106 | Educational status of your husband | 1. Illiterate (cannot read and write)  2. Literate (able to read and write)  3. Primary school – 1- 4 grade  4. Primary school – 5- 8 grade  5. Secondary school  6. College diploma and above |  |
| 107 | What is your partner’s occupation | - - - 1. Farmer       2. Government Employed       3. Daily laborer       4. Merchant       5. Student       6. Others[specify]____________ |  |
| 108 | Estimated Household income per month | ________________ETB/Month |  |
| 109 | What is the number of people who live usually in this household? | ___________________ |  |
| 110 | Do you have radio/TV in your home? | 1. Yes  2. No |  |

**II. Obstetric characteristics and maternal health care service practice**

| **S/N** | **Questions** | **Responses** | **Remark** | |
| --- | --- | --- | --- | --- |
| 201 | What is your age at your first pregnancy? | ______________ years |  | |
| 202 | How many pregnancies have you ever had? | 1. Pregnancy ____________ 2. Number of Abortion:________ 3. Number of live birth _________ 4. Number of still birth:_____ |  | |
| 203 | How many living male and female children do you have? (Express in no) | ______ male _________ female |  | |
| 204 | How many deliveries have you had in the last 2 years? | ___________ deliveries |  | |
| 205 | How long was your last delivery? | __________months |  | |
| 206 | What is the birth order of your last delivery | _________ |  | |
| 207 | Would you like to have children in the future? | 1. Yes 2. No | **If no skip to Q 209** | |
| 208 | If yes, for Q 207 how many? (Express in No) | 1. No of children desired -------- 2. Don’t know |  | |
| 209 | Do your husband/ partner want to have more children in the future? | 1. Yes 2. No 3. Don’t know |  | |
| 210 | Who is responsible for deciding to have children in your family? | 1. Wife 2. Husband 3. Joint discussion 4. Other specify_________________ |  | |
| 211 | Do you know dangerous health problems related to pregnancy? | 1. Yes 2. No | **If no skip to Q 213** | |
| 212 | If yes for Q211, can you mention some of them? (**More than one answer is possible**) | 1. Vaginal bleeding 2. Severe Headache 3. Severe abdominal pain 4. Drowsiness 5. Facia swelling 6. Hand swelling 7. Persistent vomiting 8. Others (specify)__________ |  | |
| 213 | Have you had any health related problems during last pregnancy? | 1. Yes 2. No | **If no skip to Q 301** | |
| 214 | If “Yes” to Q 213, Which of the following Problems?(Multiple response is possible) | 1. Vaginal bleeding  2.Severe Headache  3. Severe abdominal pain  4. Drowsiness  5. Facia swelling  6. Hand swelling  7. Persistent vomiting  8. Others (specify)__________ |  | |
| **Part III: Knowledge and practice on antenatal Care Service** | | | | |
| S.No | **Questions** | **Responses** | | **Remark** |
| 301 | Have you ever heard about ANC service? | 1. Yes 2. No | | **If no skip to Q 401** |
| 302 | Where do you here about the sources of ANC services? | 1. Health institution 2. Radio/TV 3. Health care provider 4. Family/Relatives 5. Friends 6. Other(Specify): ______________ | |  |
| 303 | Do you know that ANC has an advantage? | 1. Yes 2. No | | **If no skip to Q 305** |
| 304 | If “Yes” to Q303 Which of the following advantages of ANC do you know? | 1. To detect and treat health problems during pregnancy 2. To get information where to deliver 3. To check conditions of fetus 4. others, specify__________ | |  |
| 305 | Have you attended ANC for your last pregnancy? | 1. Yes 2. No | | **If no skip to Q 312** |
| 306 | How many ANC visits you had during your last pregnancy? | ______Write the number of visits. | |  |
| 307 | At what gestation age was your first visit? | _____________ month | |  |
| 308 | Why you decide to start [begin] the follow up at this time? (**More than one answer is possible**) | 1. I perceive it is appropriate time 2. From my previous Experience 3. Due to illness 4. To assure pregnancy 5. Busy by other works 6. Economic factor [money constraints] 7. Because of unplanned pregnancy 8. Others [specify]_____________ | |  |
| 309 | Where were you attended? | 1. At hospital 2. At health center 3. At private clinic 4. At health post | |  |
| 310 | Would you paid for ANC service | 1. Yes 2. No | |  |
| 311 | Have you given information to deliver in health facilities? | 1. Yes 2. No | |  |
| 312 | If “No” to Q305, Why didn’t you attend ANC visit? | 1. I don’t know about ANC 2. No problems encountered 3. Health institution was too far 4. Husband disapproval 5. No transportation 6. Can’t pay for transportation 7. Influence of other peoples 8. Fear of lack of privacy 9. Other (specify)_____________ | |  |
| 313 | Would you attend ANC if pregnant in the future? | Yes  No | |  |
| **Part IV : Knowledge and practice on delivery** | | | | |
| **S. No** | **Questions** | **Responses** | | **Remark** |
| 401 | Have you ever heard about institutional delivery service? | 1. Yes 2. No | | **If no skip to Q 501** |
| 402 | Where do you here about the sources of institutional delivery services? | 1. Health institution 2. Radio/TV 3. Health care provider 4. Family/Relatives 5. Friends 6. Other(Specify): ______________ | |  |
| 403 | Do you know a health problem that can occur during childbirth? | 1. Yes 2. No | | **If no skip to Q 406** |
| 404 | If “yes” to **Q403**, which of the following problems do you know? **(Multiple answers are possible)** | 1. Severe bleeding 2. Obstructed labour 3. Fetal death 4. Maternal death 5. Others, specify______________ | |  |
| 405 | Do you know that the above problems and their outcome are manageable by institutional delivery? | 1. Yes 2. No | |  |
| 406 | Where did you deliver your last child? | 1. In health facilities 2. At home | | **If at home skip to Q 408** |
| 407 | If your response to **Q405** is “at health facility”, Why you preferred to deliver in health facility? **(Multiple answers are possible)** | 1. Because of my previous bad experience from home delivery 2. I was informed to deliver in health Facilities 3. I have faced obstetric problems which forced me to deliver in health facility 4. Others (Specify)___________ | |  |
| 408 | Why you preferred home delivery? | 1. The labour was going well 2. I feel more comfortable at home 3. Close attention from relatives & family numbers 4. It is my usual practice 5. Previous bad experience from ID 6. Cannot afford to pay for health services 7. No transportation services 8. Cannot pay for transportation services 9. My husband decision 10. Other reasons, specify______ | |  |
| 409 | Have you faced any health problems immediately after delivery? | 1. No  2. Yes, specify them ----------------- | |  |
| 410 | Who made the final decision about your place of last delivery? | 1. Just me  2. My husband  3. My relatives  4. Other people, specify__________ | |  |
| 411 | Where do you want to deliver if you become pregnant in the future? | 1. Health institution 2. Home | | **If health institution skip to Q 501** |
| 412 | If you intend to deliver at home, would you tell me the main reasons? **(Multiple answers are possible)** | 1. I feel more comfortable at home 2. Close attention from relatives & family numbers 3. It is my usual practice 4. Previous bad experience from ID 5. Cannot afford to pay for health services 6. No transportation services 7. Cannot pay for transportation services 8. My husband disapproval 9. Religious prohibition 10. Other reasons (specify) ______ | |  |

**Questions on Postnatal care service practice**

| **S/N** | **Questions** | **Responses** | **Remark** |
| --- | --- | --- | --- |
| 501 | Have you ever heard about PNC service? | 1. Yes 2. No | **If no skip to Q 601** |
| 502 | Where do you here about the sources of PNC services? | 1. Health institution 2. Radio/TV 3. Health care provider 4. Family/Relatives 5. Friends 6. Other(Specify): ______________ |  |
| 503 | Do you know that PNC service has an advantage? | 1. Yes 2. No | **If no skip to Q 505** |
| 504 | If “Yes” to **Q503**, Which of the following advantages of PNC do you know? (**Multiple answer is possible**) | 1. To detect and treat health problems during postpartum period 2. To get information how to feed their infant 3. To give opportunity for family planning 4. To check conditions of infants 5. Others (specify)__________ |  |
| 505 | Do you know a health problem that can occur during postnatal period? | 1. Yes 2. No | **If no skip to Q 507** |
| 506 | If “yes” to **Q505**, which of the following problems do you know? (**Multiple answer is possible**) | 1. Bleeding (PPH) 2. Sepsis 3. Peurperal psychosis 4. Maternal death 5. Others, specify____________ |  |
| 507 | Did you attend postnatal services in the six weeks after delivery? | 1. Yes  2. No | **If “No” Skip to Q512** |
| 508 | If “Yes” to **Q 507** What postnatal services did you receive when you went back to hospital after delivery? | 1. Physical examination 2. Immunisation of baby 3. Counselling 4. Family planning services 5. Breast feeding education 6. Other (specify)___________________   _________________________________ |  |
| 509 | How many PNC visits you had during your last pregnancy? | ______Write the number of visits. |  |
| 510 | At what time was your first visit? | _________ hours  _________ days |  |
| 511 | Why did you go for postnatal services?  (**More than one could be marked if applicable**) | 1. Because was ill 2. Because the baby needed it’s immunisation 3. Because the midwife had told me I should 4. Because I wanted to start family planning 5. Because I wanted to make sure I am back to 6. Other (specify)………………… |  |
| 512 | If “No” to Q507, What were the factors that prevented you from attending postnatal services?(**Multiple response is possible**) | Health professional shouted at me  They did not teach me well  Examined me roughly  Waiting more time at the facility  Religious forbidden  ignorance of my privacy  Other (specify)…………….. |  |
| 513 | Would you like to attend postnatal care if you become pregnant in the future? | 1. Yes 2. No |  |

**Part V: Questions on knowledge and practice of family planning service practice**

| **S/N** | **Questions** | **Responses** | **Remark** |
| --- | --- | --- | --- |
| 601 | Have you ever heard family planning methods? | 1. Yes 2. No | **If no stop here** |
| 602 | If yes for **Q 601**, what are the sources of information for modern contraception? (More than two answer possible) | 1. Health Worker 2. Radio 3. TV 4. Friends 5. Other (specify)----------------------- |  |
| 603 | If yes for **Q 601**, What type of modern contraceptive methods do you know? (Read and thick all mentioned  Method   1. Pill 2. Injectables 3. IUDs 4. Implants/Norplant 5. Spermicidal 6. Condom 7. Female sterilization 8. Male sterilization | Yes No  ____ ____  ____ ____  ____ ____  ____ ____  ____ ____  ____ ____  ____ ____  ____ ____ |  |
| 604 | If yes for **Q601** What general uses of family planning methods do you know? | 1. Improve maternal health 2. Improve child health 3. Increase wealth of the family 4. Increase wealth and prospective of the community 5. Increase national economic growth 6. Other specify --------------------------- |  |
| 605 | Have you ever discussed with health professional about family planning methods? | 1. Yes 2. No |  |
| 606 | Have you ever discussed with your husband about family planning methods? | Yes  No |  |
| 607 | Did you ever use family planning methods? | 1. Yes 2. No | **If no skip to Q 612** |
| 608 | If “yes”**607** which method of family planning methods do you ever used?   1. Pill 2. Injectables 3. IUDs 4. Implants/Norplant 5. Spermicidal 6. Condom 7. Female sterilization 8. Male sterilization | Yes No  ____ ____  ____ ____  ____ ____  ____ ____  ____ ____  ____ ____  ____ ____  ____ ____ |  |
| 609 | Are you Currently using any FP method | 1. Yes 2. No | **If no skip to Q 613** |
| 610 | If “Yes” for **Q 609**, which method of family planning method are you using currently**?** | 1. Pill 2. Injectables 3. IUDs 4. Implants/Norplant 5. Spermicidal 6. Condom 7. Female sterilization 8. Male sterilization |  |
| 611 | Why do you use family planning services? (**More than one could be marked if applicable**) | 1. To prevent unwanted pregnancy 2. To space the birth interval 3. Because of having enough children 4. Because of disease condition 5. Because I wanted to promote the health of born children 6. Because shortage of income 7. Other (specify)………………………. |  |
| 612 | If you never used family planning methods, what were the reasons? (**More than one answer is possible**) | 1. Fear of side effect 2. Lack of awareness of the family planning methods 3. Rumors they are not good 4. Important others influence 5. To have more children 6. Husband disapproval 7. Religion prohibition 8. Fear of infertility 9. Other **(specify)_________________**____ |  |
| 613 | Would you like to use family planning methods in the future? | 1. Yes 2. No 3. Don’t know | **If no/don’t know skip to Q615** |
| 614 | If yes for question Q 613, specify the method of LAMPs you intend to use? (**More than one answer is possible**) | 1. Pill 2. Injectable 3. IUDs 4. Implants/Norplant 5. Spermicidal 6. Condom 7. Female sterilization 8. Male sterilization |  |
| 615 | If you are not intending to use family planning methods, would you tell me the main reasons? | 1. Fear of side effect 2. Lack of awareness of the family planning methods 3. Little risk of pregnancy 4. To have more children 5. Husband disapproval 6. Religion prohibition 7. Fear of infertility 8. Other **(specify)_________________** |  |

**Now I have completed my questions thank you for your cooperation.**
